# The role of immune checkpoints PD-1 and CTLA-4 in cardiovascular complications leading to heart failure

**DOI:** 10.3389/fimmu.2025.1561968

**Published:** 2025-04-04

**Authors:** Shoulian Huang, Yu Kang, Ting Liu, Yan Xiong, Zixuan Yang, Qing Zhang

**Affiliations:** ^1^ Department of Cardiology, West China Hospital, Sichuan University, Chengdu, Sichuan, China; ^2^ Department of Cardiology, The Second People’s Hospital of Yibin, Yibin, Sichuan, China; ^3^ Institute of Cardiovascular Diseases & Department of Cardiology, Sichuan Provincial People’s Hospital, School of Medicine, University of Electronic Science and Technology of China, Chengdu, China

**Keywords:** atherosclerosis, myocarditis, programmed cell death protein 1 (PD-1), cytotoxic T-lymphocyte-associated protein-4 (CTLA-4), cardiotoxicity

## Abstract

Immune checkpoints, such as PD-1 and CTLA-4, are crucial regulators of immune responses, acting as gatekeepers to balance immunity against foreign antigens and self-tolerance. These checkpoints play a key role in maintaining cardiac homeostasis by preventing immune-mediated damage to critical organs like the heart. In this study, we explored the involvement of PD-1 and CTLA-4 in cardiovascular complications, particularly atherosclerosis and myocarditis, which can lead to heart failure. We conducted a comprehensive analysis using animal models and clinical data to assess the effects of immune checkpoint inhibition on cardiac function. Our findings indicate that disruption of PD-1 and CTLA-4 pathways exacerbates myocardial inflammation, accelerates atherosclerotic plaque formation, and promotes the development of heart failure. Additionally, we observed that immune checkpoint inhibition in these models led to increased infiltration of T lymphocytes, higher levels of pro-inflammatory cytokines, and enhanced tissue damage. These results suggest that PD-1 and CTLA-4 are critical in preserving cardiac health, and their inhibition can result in severe cardiovascular toxicity. Our study emphasizes the need for careful monitoring of cardiovascular health in patients undergoing immune checkpoint inhibitor therapies.

## 1 Introduction

The inflammation in the human body is modulated via immune checkpoints that facilitate the communication between immune and non-immune cells and can either activate or attenuate the immune response (1). Thus, immune checkpoints are the key drivers of adaptive immunity that act as gatekeepers to maintain the immune homeostasis in the body and keep autoimmunity at bay (2, 3). Immune checkpoints are an assembly of proteins expressed on the cellular facet of Thymocytes and antigen-presenting cells that act as corresponding receptors and ligands for each other (4). Immune checkpoints regulate the process of self-tolerance by governing the intensity and efficacy of Thymocytes, thus shielding tissues and organs from inflammatory immune action (5). Immune checkpoints comprise of both stimulatory as well as inhibitory signaling pathways and a balance in these pathways helps to maintain the immune homeostasis required for self-tolerance (2). The stimulatory signals activate the immune system to act while the inhibitory signals are required to halt the immune response and thus preventing self-tissue destruction (6).

The antigen-specific immune response is referred to as adaptive immunity. In this type of immune response, the antigen is presented, recognized and processed resulting in the production of an excessive number of immune cells that invade the antigen. Simultaneously, ‘memory’ cells are generated that help in efficient response against antigens in future. CD8+ cytotoxic T cells and CD4+ helper T cells are the major cells involved in acquired immunity (7). Activation of T cells occurs in three steps (1, 7–10)-

- Binding of T cells to Major Histocompatibility Complex (MHC)-bound peptides on Antigen-Presenting Cells (APCs): T-cell activation begins when the T-cell receptor (TCR) on a naïve T cell binds to the peptide-MHC complex presented on the surface of an antigen-presenting cell (APC), such as a dendritic cell. This interaction is highly specific; the TCR recognizes a unique peptide presented on MHC molecules, which are either Class I (for CD8+ cytotoxic T cells) or Class II (for CD4+ helper T cells). This initial binding is crucial for the recognition of foreign antigens and for initiating the immune response.
- Co-stimulatory Signal to Prevent Anergy: In addition to the TCR-peptide-MHC binding, a second signal is required for full T-cell activation. This co-stimulatory signal typically involves the interaction of CD28 on the T cell with its ligands, CD80 or CD86, on the APC. This interaction prevents T-cell anergy, which is a state of unresponsiveness that occurs when T cells are exposed to antigen in the absence of appropriate co-stimulation. Without this co-stimulatory signal, the T cell may become tolerant to the antigen, thereby avoiding an autoimmune response.
- Cytokine Signaling to Modulate Immune Response: Upon successful engagement of both the TCR and co-stimulatory receptors, APCs release various cytokines that play a critical role in shaping the intensity and quality of the immune response. These cytokines, such as interleukin-2 (IL-2), promote T-cell proliferation, differentiation, and survival. They also help determine whether the activated T cells become effector cells (such as Th1, Th2, or Th17 cells) or regulatory T cells (Tregs), each of which plays a distinct role in the immune response. Cytokines influence the immune response by modulating the differentiation of T cells into specific subsets that either promote inflammation or suppress it to maintain immune homeostasis.

Multiple interactions between Thymocytes and APCs, stimulatory or inhibitory act as a checkpoint that moderates Thymocytes immune reaction and immuno-homeostasis. The co-stimulatory or co-inhibitory signals are proteins that can either activate (turn up) or inhibit (turn down) the immune cell response and are referred as immune checkpoint proteins (11).

The body maintains a balance between immune response against antigens and normal body tissues and organs. Certain body parts like the eyes, brain and heart, are safeguarded from the inflammatory immune response by specific regulatory mechanisms. Healthy human myocardial cells possess a small set of Thymocytes and have regulatory mechanisms that prevent the recruitment and activation of Thymocytes and memory Thymocytes. However, for maintaining and regulating tissue homeostasis and the repair mechanism of tissue, dendritic cells and macrophages are found in the myocardium wherein, these cells have potential of initiating effector naïve Thymocytes (12). Additionally, the richly supplied microvasculature of the heart not only provides oxygen and nutrients to the heart cells but also aid in supplying circulating Thymocytes to the heart. The present review will emphasize understanding these regulatory immune mechanisms in the heart that help to protect the cardiac from the immune response. The current review will focus on understanding various vascular and cardiac immune checkpoints involved in cardiac homeostasis and their role and mechanism involved in numerous cardiovascular complications that can incite heart failure like, atherosclerosis and myocarditis.

Immune checkpoints, such as PD-1 and CTLA-4, are pivotal in regulating these immune processes within the cardiovascular system. These checkpoints control the activation, proliferation, and exhaustion of immune cells, ensuring that inflammation does not become excessive and that self-tolerance is maintained. Dysregulation of immune checkpoint pathways can lead to aberrant immune responses, contributing to cardiovascular diseases such as atherosclerosis and myocarditis. In atherosclerosis, immune checkpoint inhibition can exacerbate inflammation and promote plaque instability, while in myocarditis, the unchecked activation of immune cells can lead to myocardial injury and fibrosis. This review focuses on understanding the mechanisms by which immune checkpoints influence cardiovascular homeostasis and their involvement in the development of heart failure through conditions like atherosclerosis and myocarditis.

## 2 Inflammatory landscape in cardiovascular complications

The association amid inflammation and cardiovascular diseases has been recognized and adaptive immunity pertaining to Thymocytes and its activation have been found to be instrumental in cardiovascular diseases (13–16). Chronic inflammation in the arteries propels the progression of atherosclerosis. Atherosclerosis is the predominant driver of cardiovascular complications and is marked by the growth of plaques in the arterial walls. The inflammation activates immune cells that induce the secretion of cytokines as well as chemokines, which in turn promotes the accretion of immune cells at the inflammation site (17, 18). Endothelial dysfunction also promotes cardiovascular complications wherein factors like hypertension and elevated levels of cholesterol predispose arterial walls to the enhanced infiltration and accumulation of macrophages and monocytes. Immune cells i.e., macrophages promote the formation of foam cells by engulfing oxidized oxidized low-density lipoproteins. Foam cells are the initial drivers of plaque formation or atherosclerosis by inducing the development of lipidic streaks in the artery wall (19–22). Apart from macrophages, Thymocytes are the predominant player in cardiovascular complications. CD4^+^ T cells or helper Thymocytes modify into different subsets with specialized functionality. T-helper 1 or Th1 cells secrete pro-inflammatory cytokines like interferon (IF)-γ, which is a promoter of inflammation and destabilizer of plaque (23–25). While T-helper 2 or Th2 cells induce the production of anti-inflammatory cytokines viz., interleukin (IL)-4, 10, which hinders the progression of plaque. T-helper 17 or Th17 cells have been associated with prolonged inflammation and increased risk for plaque formation (26). Regulatory cells or Treg cells halt inflammation and stabilize the plaque (27). In the heart, natural killer cells (Nk cells) exhibit cytotoxic activity and induce pro-inflammatory cytokines production which promotes the progression and destabilization of plaques (28). Alternatively, B-cells, also play a key role in systemic inflammation and the advancement of atherosclerosis (29).

Chronic inflammation marked with distinctly high levels of pro-inflammatory myocardial and systemic cytokines alters the left ventricular function and induces its remodeling as well (30, 31). The lack of harmony between inflammation-promoting cytokines and anti-inflammatory cytokines contributes to heart failure (32). Amplified pro-inflammatory cytokines has been found to be linked with heart failure (33). Inflammatory cytokines that have been found to be associated with cardiac complications comprise IL-1, 6, 8, 18, 33 and IL-1RA (34). Inflammatory cytokines, primarily released by neutrophils, induce cardiac remodeling and alter cardiac function by assisting in inducing apoptosis of cardiomyocytes and activating the matrix metalloproteinase (35, 36). Activation of IL-18 has been found to be related to an elevated risk for cardiovascular diseases with hospitalization risk, especially in patients with congestive heart failure (37). Similarly, a higher amount of IL-6 has been correlated with an amplified rate of hospitalization in patients with heart failure and even mortality (38). Cytokine IL-8 is also positively correlated with heart failure risk and hospitalization as compared to normal patients (36, 39, 40). Higher levels of cytokines IL-1RA are found in heart failure with reduced ejection fraction, irrespective of the diabetic status of the patients (40). IL-1β endorses infiltration of leucocytes at the sites of injury and has been found to be correlated with cardiac events promoting and leading to heart failure (41). On the contrary, levels of cytokine IL-33 are reduced in patients with heart failure as against healthy subjects (41).

Galectin (Gal)-3, a lectin of galectin family, is implicated in the signaling of different intracellular pathways of inflammation, cellular migration, immune response and signaling, by binding with intra-cellular as well as surface ligands (42). Cardiac remodeling has been found to be associated with Gal-3 and is considered a bio-signature of fibrosis and heart failure. Gal-3 is associated with inducing a signaling pathway for transforming growth factor-β SMAD3 and thus resulting in induction of collagen production, proliferation of myofibroblast, cardiac hypertrophy and infiltration of macrophages (43–46). A clinical study findings suggest Gal-3 to be a positive indicator of heart failure-related mortality in the follow-through period of 6.5 years (47). Further, in this clinical evaluation, Gal-3 was also found to be positively associated with left ventricular end-diastolic volume enhancement (48).

The necrotic cells secrete injury-associated molecular signatures i.e., heat shock protein 60 or HSP 60 and high-mobility group box 1 (HMGB1) protein, in amplified amounts, in case of cardiac stress, hypertension, metabolic syndrome and ischemic injury. These proteins are identified by natural immune cells via pattern recognition receptors. Consequently, the secretion of pro-inflammatory cytokines by M1 macrophages and non-immune cells which initiates the deployment of phagocytic cells of the immune system, removal of apoptotic cells, and renewal of tissues (49). C-reactive protein (CRP), IL-6, 1β and TNF-α expressed by innate immune cells upon inflammation have been associated with myocardial infarction, coronary disease and stroke (50). Increased vascular permeability and reduced amount of nitric oxide is associated with endothelial dysfunction. Pro-inflammatory cytokines released by macrophages promote rapid growth of mural cells and aggregation of particles of oxycholesterol-containing low-density lipoprotein (LDL) in vessels (51). Macrophages M1 is found to be associated with atherosclerotic plaque leading to acute coronary syndrome. Cytokines IL-6, 12, reactive nitrogen species and reactive oxygen species are pro-atherosclerotic as they increase the oxidative stress in atherosclerosis. The carotid artery plaques and its progression have been found to be linked with amplified levels of Th1, Th17 and IL-17, 23, 21 (52). Senescent CD14^+^CD16^+^ monocytes and CD4^+^ effector memory T cells (CD3^+^CD4^+^CD45RA^−^CD45RO^+^CCR7^−^) are found in atherosclerotic plaques (49, 53). While, late-differentiated CD4^+^CD28^−^ peripheral Thymocytes have been linked with acute coronary events (54).

## 3 Immune cell activation pathways

Originating from the thymus, matured naïve T lymphocytes (both CD8+ and CD+4) reach the secondary lymphatic organs via systemic circulation. Systemic circulation also helps in the accumulation of proteins and antigens.

Activation of T-lymphocytes can occur in two ways, one of them being activation mediated via dendritic cells. These antigens are then presented to the T-lymphocytes as peptide-MHC complexes expressed on the facet of antigen-presenting cells. The dendritic cells in the secondary lymphatic organs are the antigen-presenting cells that are critical for the activation of naïve T lymphocytes, However, the presentation of antigens by the antigen-presenting cells must be accompanied by the presence of tissue injury or pathogen, i.e., danger signals, for dendritic cells to express the co-stimulatory surface ligands. Thus, activation of T-lymphocytes occurs only when both the steps are present i.e., antigen presentation by the antigen-presenting cell, in combination with expression of co-stimulatory ligands on the facet of dendritic cells as a response to antigens or injury. Few costimulatory proteins for naïve T cells are from the immune serum globulin superfamily which includes, B7-1 (CD80) and B7-2 (CD86). The CD80 and CD86 are found on dendritic cells having prior exposure to pathogens as well as innate immune stimuli. These two synchronized steps result in the activation of naïve T lymphocytes prompting modification of Thymocytes to cytotoxic CD8+ or effector CD+4 helper cells. Thus, costimulatory signals resulting in immunological synapse formation of Thymocytes are limited to conditions wherein there are specific risks involved. Further, for functional activation, effector Thymocytes identify the antigen at the place of inflammation (55).

The alternative method of activation of T-lymphocytes is not dependent upon dendritic cells wherein antigen MHC presentation is carried out by cells other than dendritic cells. Also, in this case, the co-stimulation step is not pertinent for the immunological synapse formation of Thymocytes. In this approach, CD275 is an inducible thymocyte co-stimulator which is a part of an immunoglobulin superfamily found on B- cells and binds with the corresponding CD278 expressed on CD+4 T helper cells. The alternative costimulatory pathway also includes the binding of tumor necrosis factor protein receptors found on Thymocytes with corresponding tumor necrosis factor protein found on APCs, for e.g., for the stimulation of CD8+thymocyte response, ligand found on APCs i.e., 4-1BB ligand or 4-1BBL binds with 4-1BB or CD137 found on thymocytes. Similarly, another ligand found on antigen-presenting cells is CD252 or OX40 ligand interacts with CD134 or OX40 found on thymocytes (56, 57).

Apart from the above mechanisms, there are an additional mechanism that limits the activity of thymocytes and imparts the capability for self-tolerance toward its own cells/tissues. The mechanism that suppresses the action of thymocytes via programmed cell protein death-1 (PD-1) or CD279 and cytotoxic T-lymphocyte-associated protein-4 (CTLA-4) or CD152. These proteins are homologous to CD28 structurally but functionally contradictory i.e., PD-1 and CTLA-4 inhibit the functioning of thymocytes. Immediately after activation of thymocytes, CTLA-4 is found on the cellular facet of thymocytes, including naïve Thymocytes, regulatory T cells (Treg) and memory Thymocytes. However, Treg and memory Thymocytes express CTLA-4 when exposed chronically to antigens which results in Thymocyte exhaustion (58). The CTLA-4 expression is antigen exposure dependent and is expressed highly on exhausted thymocytes. The CTLA-4 competitively contests with CD28 to bind with B7, thus hampering the B7-CD28-dependent activation of T-lymphocytes (58, 59).

PD-1 or CD274/PD-2 or CD273 expression on T lymphocytes is initiated by the exposure to antigen. PD-1 and PD-2 are part of the immune serum globulin superfamily and are structurally homologous to CD28. The expression of ligands for PD-1 i.e., PD-L1, dendritic cell, epithelial parenchymal, endothelial cells and macrophages, is induced by the cytokines interferon-gamma (IFN-γ) and type-1 IFNs, while PD-L2 is found only on bone-marrow derived antigen-presenting cells (60, 61). Consequently, the binding of CD28 and thymocyte receptors activates the protein tyrosine kinases pathway, however, the binding of PD-1 and PD-L1 results in the deployment of protein tyrosine kinases, which hinders the protein tyrosine kinases pathway and suppresses thymocyte activity. Thus, both PD-1 and CTLA-4 shuts off the antigen-activated co-stimulatory thymocyte activation, thus PD-1 and CTLA-4 are referred to as co-inhibitors or immune checkpoints (62).

Apart from PD-1 and CTLA-4, other immune checkpoints are, Lymphocyte activation gene-3 (LAG-3) or CD223. LAG-3 also belongs to the immune serum globulin superfamily and is found on thymocytes, B-cells, macrophages and dendritic cells. LAG-3 inhibits the antigen presentation by binding with the class II MHC molecules (63–65). Another member of the immune serum globulin superfamily, which is found on natural killer (NK) cell, Thymocyte and macrophages, is the T cell immunoglobulin-3 (TIM-3) and inhibits the thymocyte activation by binding with carcinoembryonic antigen-related cell adhesion molecule-1 (CEACAM-1), galectin-9, and phosphatidyl serine (66–69). Another immune checkpoint is the thymocyte immune receptor containing Ig and ITIM domains (TIGIT), found upregulated in thymocytes and NK cells that bind with CD112 and CD155. TIGIT, LAG-3 and TIM-3 are found in amplified levels on the exhausted T cells (70, 71).

Another important component for self-tolerance and non-susceptibility of commensal microbes are the Treg cells, of which CD4^+^CD25^+^FoxP3^+^ Thymocytes are most widely studied. Studies show that mutation in FoxP3 can incite autoimmune diseases (72). Treg cells express PD-1, LAG-3 and CTLA-4 immune checkpoints (73, 74).

## 4 Immune checkpoints involved in cardiac homeostasis

### 4.1 Cytotoxic T lymphocyte-associated 4 (CTLA-4) protein

CTLA-4 is a surface receptor found on thymocytes (primarily on activated CD4+thymocytes, CD8+thymocytes and Treg cells), which is negatively linked with thymocyte activation and thus enforces preservation of cardiac tissues and maintains cardiac homeostasis (75). Primarily, CTLA-4 works as a regulator that controls the magnitude of activation of thymocytes (76, 77). CTLA-4 is a co-inhibitory receptor that functions in contradiction with the functioning of CD28. CD 28 is a co-stimulatory surface protein found on thymocytes which is a pre-requisite for the activation of thymocytes. The ligands for CD 28 are CD 80/86 or B-7 1/2 found on the cellular facet of antigen-presenting cells. The linkage between CD 28 and CD 80/86 incites the secretion of cytokines and cellular proliferation (78). CTLA-4 is structurally similar to CD28 and has a common affinity toward CD80/86, although CTLA-4 has a higher affinity to CD 80/86, thus CTLA-4 competitively and preferentially binds to CD80/86 which suppresses the multiplication of thymocytes and production of interleukin (79–83). The CTLA-4 acts as a ‘switch off’ indicator that controls the proliferation of thymocytes. The expression of CTLA-4 occurs post-activation of Thymocytes, however, it acts as a controller that confines the proliferation of thymocytes (84, 85). The immune response of CTLA-4 is manifested on CD4+thymocyte as, an amplification of Treg cells and halts activity of Th cell (86, 87). CTLA-4 promotes cardiac protection from the immune response (88). Animal studies have shown that CTLA-4 inhibitors resulted in enhanced myocardial inflammation and eventually myocarditis (89). Lymphoproliferative disease wherein infiltration of lymphocytes in multiple organs and tissue inflammation and necrosis like, myocarditis, is seen in animal knockout models devoid of CTLA-4 (90, 91). CTLA-4 deficit even in Treg cells is capable of myocarditis induction. The absence of expression of CTLA-4 in Treg cells results in lymphoproliferation wherein infiltration of myocardial cells and impairment of myocytes. Additionally, the absence of CTLA-4 expression further exacerbates the cardiac complications (86, 92). Study findings suggest that cardiac tissues are protected considerably from the action of cytotoxic Thymocytes due to the protective effect imparted by CTLA-4 (93). These findings indicate cardiotoxic effects pertaining to the suppression of CTLA-4 (94). The protective effect of CTLA-4 has been reported in several animal studies wherein increase in mRNA of CTLA-4 has been correlated with amplification of Treg cells and reduction in atherosclerotic plaque (95–97).

### 4.2 PD-1/PDL-1 signaling

The function of preserving peripheral tolerance and fend off autoimmune response is carried out by PD-1/PD-L1 (98, 99). PD-L1 or CD274 or B7-H1, is the co-inhibitory ligand for PD-1 that controls the activity of thymocytes (100). PD-1 is found on the facet of activated thymocytes while its corresponding ligand, PD-L1 is found on antigen-presenting cells (101, 102). Latent and stimulated thymocytes, B-cells, dendritic cells, myeloid cells and immune-privileged tissues like, the placenta, brain, heart, endothelial cells, muscles and pancreas demonstrate PD-L1 while monocytes and dendritic cells solely demonstrate PD-L2 (103). PD-L1/L2 binding with PD-1 forms the basis of immune response for self-tolerance (104, 105). PD-1/L1 binding helps to restrain the excessive immune response which can induce cellular destruction and autoimmune effect. Activated PD-L1- PD-1 pathway results in suppression of effector thymocytes, which helps in preserving self-tolerance along with resolving inflammatory response (106). However, inhibition of PD-1/L1 interaction can induce an inflammatory immune response in protected tissues like cardiac tissues. Accordingly, the paucity of interaction between the PD-1/L1 pathway incites exacerbation of lesions in atherosclerosis (107). The development of cardiomyopathy in animals devoid of PD-1 also indicates the critical role of PD-1/PD-L1 in maintaining self-tolerance toward critical organs like the heart (99). Reduced levels of PD-1 and PD-L1 on thymocytes and antigen-presenting cells are observed in patients with coronary artery disease, which induces higher levels of CD4+ and CD8+ cells along with an amplified amount of pro-inflammatory cytokines (108).

### 4.3 LAG-3 and cardiac homeostasis

In addition to PD-1 and CTLA-4, LAG-3 has recently emerged as a critical immune checkpoint involved in regulating immune responses in cardiac tissues. LAG-3 functions as an inhibitory receptor that downregulates T cell activation and contributes to immune tolerance. It is expressed on activated T cells and plays a role in preventing excessive immune responses that could lead to tissue damage. In the context of cardiovascular diseases, including atherosclerosis and myocarditis, LAG-3 may serve to modulate the inflammatory response, reducing the risk of autoimmune damage to the heart. Its expression in the myocardium during inflammatory conditions suggests that LAG-3 could be a potential target for therapeutic interventions aimed at controlling immune-mediated cardiovascular injuries. Future studies are needed to further elucidate its role in cardiac homeostasis and its potential as a therapeutic target in cardiovascular diseases.

## 5 Mechanisms of cardiovascular injury

The cardiotoxic events observed in patients treated with immune checkpoint inhibitors have highlighted the pivotal role of immune checkpoints in regulating cardiovascular health (109–116). For example, myocarditis, one of the most severe immune checkpoint inhibitor-related toxicities, has been documented in cases where patients receiving anti-PD-1 therapies, such as pembrolizumab and nivolumab, developed fatal myocarditis, resulting in cardiac arrest in some instances. The inhibition of immune checkpoints led to the activation of CD8+ T cells and macrophages, which infiltrated cardiac tissue and induced inflammation, compromising myocardial function. Additionally, left ventricular dysfunction, even in the absence of myocarditis, has been observed in patients treated with CTLA-4 inhibitors like ipilimumab. This dysfunction was linked to systemic inflammation and cytokine release, which impaired cardiomyocyte contractility and promoted tissue remodeling. Vasculitis has also been seen in patients treated with immune checkpoint inhibitors, as exemplified by a case where nivolumab-induced acute coronary vasculitis led to lymphocyte infiltration in the coronary vessels, causing endothelial dysfunction and plaque rupture. Lastly, pericarditis has been reported in patients, presenting with chest pain and fluid accumulation around the heart, which resulted from inflammation in the pericardium following immune checkpoint activation. These cases emphasize the complex mechanisms through which immune checkpoint inhibition can drive cardiovascular injury, underscoring the need for careful monitoring and management of cardiovascular health in patients receiving such therapies.

### 5.1 Immune checkpoints aided cardiac injury

The unrestrained activity of the immune cells pertaining to association with the cardiac tissues, especially post-immune checkpoint inhibitor therapy can drive cardiac injury. Inhibition of immune checkpoint inhibitors drives an attack of lymphocytes on the normal, non-antigenic and non-cancerous tissues and cells of the body. Cardiac lymphocytic infiltration leading to myocarditis was observed in an animal study wherein mice were deficient of CTLA-4. A massive upsurge in levels IL-4, colony-stimulating factor and IFN-γ was observed in CTLA-4 deficient mice as against normal mice, which eventually resulted in the induction of lethal myocarditis in animals. Thus indicating the criticality of CTLA-4 in maintaining cardiac immune equilibrium. The findings of this study also depict the importance of estimating the levels of these cytokines as a prognostic indicator of myocarditis, especially in patients getting immune checkpoint inhibitor therapy (90).

**Figure 1:**
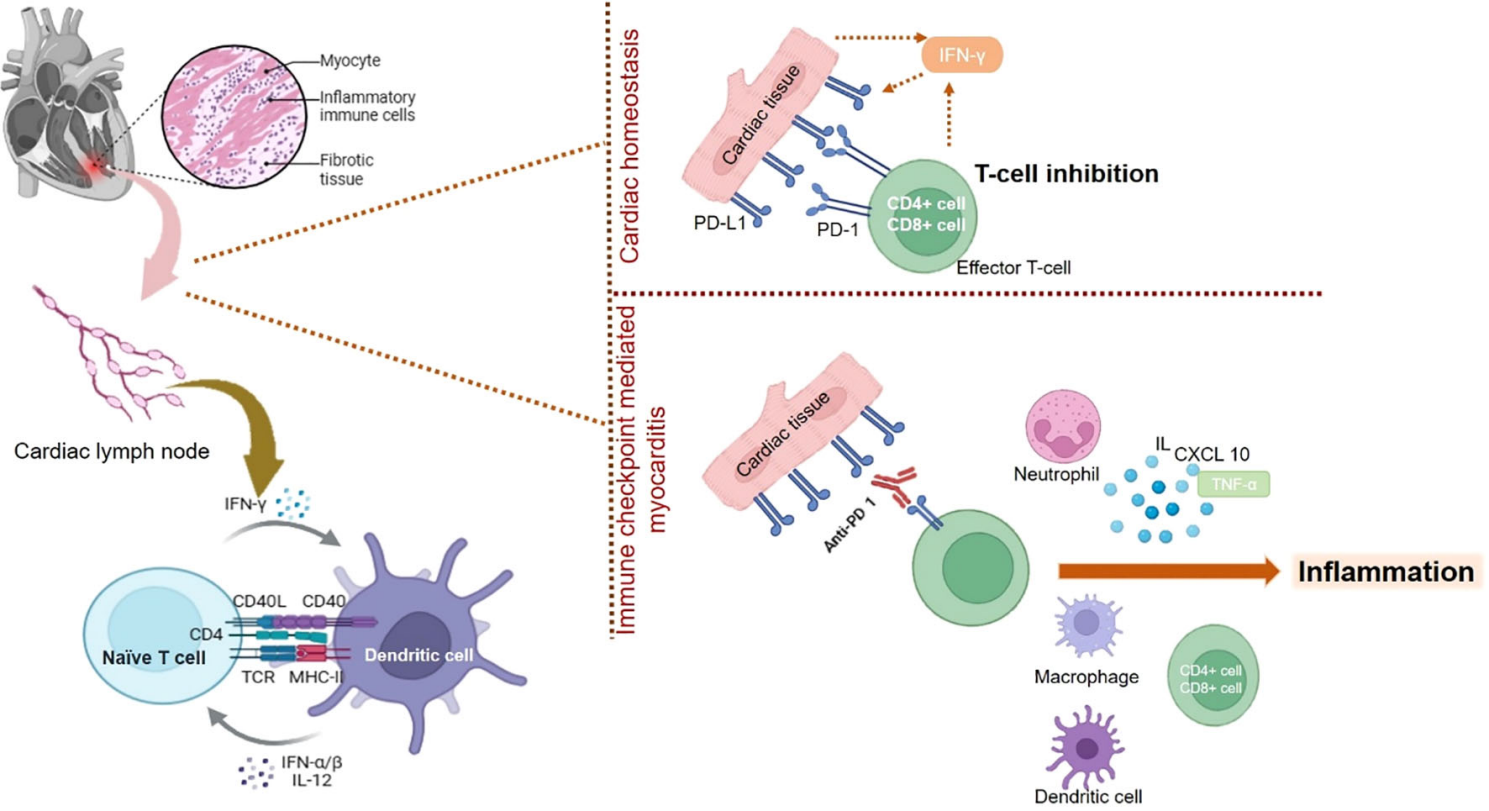
The mechanistic role played by PD-1/L1 in maintaining cardiac homeostasis and its inhibition results in immune checkpoint mediated myocarditis. Blockade of PD-1/L1 disrupts the cardiac homeostasis resulting activation of thymocytes or T-cells, which results in expression of pro-inflammatory condition leading to myocarditis.

Apart from CTLA-4, other immune checkpoints like myocardial PD-1 have also been found to affect cardiac homeostasis. The PD-1 immune checkpoint inhibit immune response mediated inflammatory response in cardiac tissues via increased expression of GADD153 (117, 118). PD-1 The animals that were devoid of PD-1 were found to develop autoimmune-based dilated cardiomyopathy wherein a significant amount of auto-immune antibodies targeted toward cardiomyocytes (119). The subjects treated with PD-1 inhibitors that developed myocarditis were found to have a significant amount of infiltrating thymocytes and macrophages similar to that found in the tumors as well as skeletal muscle cells (119). The role of PD-1 in cardiac injury was further confirmed by a study wherein IFN-γ mediated increase in PD-1 resulted in cardioprotective function. This also confirms that loss in PD-1 can drive cardiac injury (120). Loss or deficiency in the levels of PD-1 induces a rise in the levels of cardio-toxic cytokines resulting in cardiac injury as seen in the infarct area post reperfused acute myocardial infarction (117). In an animal study, wherein the animals were deficient in PD-1, exhibited the development of autoimmune myocarditis. The animals were found to have excessive levels of infiltrating myeloid cells, CD8^+^ and CD4^+^ thymocytes in the cardiac tissues along with elevated levels of auto-immune antibodies against cardiac myosin (121). A diagramatic representation of involvement of PD-1/PD-L1 in cardiac homeostasis and cardiac injury is shown in **Figure 1**.

### 5.2 Immune checkpoints aided vascular injury

**Figure 2:**
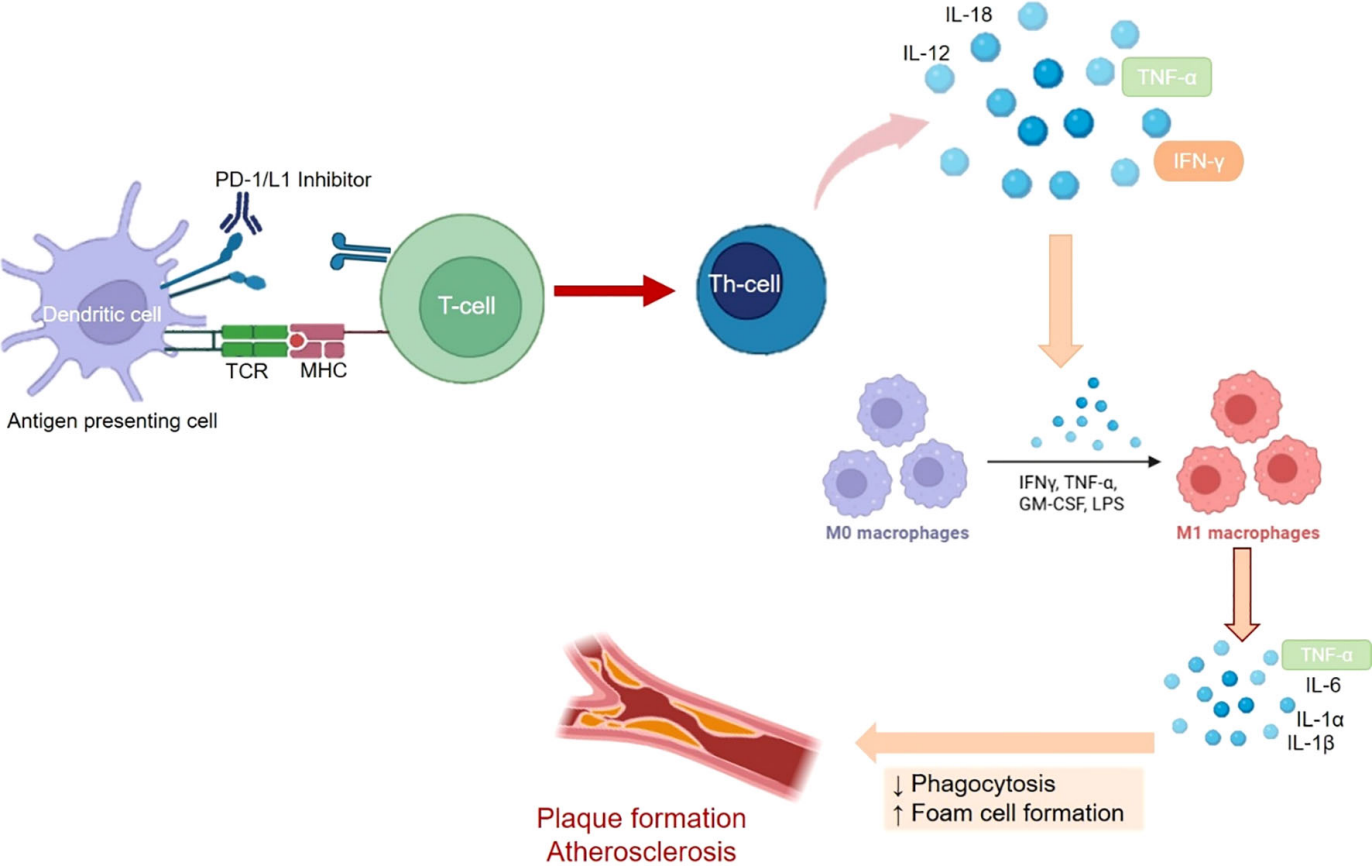
The mechanistic role of played by PD-1/L1 in immune system mediated development of Atheroscelrosis. Blocking of PD-1/L1 results in activation of thymoctes or T-cells resulting in expression of cytokines like IFN-γ, ILs and TNF-α. This further stimulates the differentiation of macrophages and subsequent release of cytokines like, TNF-α and Ils, which promote the development and proliferation of atherosclerotic plaque.

Immune checkpoint-related cardiovascular events also involve vascular injury and subjects having a higher neutrophils to lymphocyte (NLR) ratio are at higher risk of developing vascular injury leading to cardiovascular events. However, levels of C-reactive protein were found to be unrelated to the incidence of vascular injury (122). The most prominent role of immune checkpoints in vascular injury is pertaining to the advancement of atherosclerosis. Deposition of lipidic cells beneath the endothelium of large arteries is referred to as Atherosclerosis (123). In atherosclerosis, under the influence of NF-κB and with the help of adhesion molecules like, vascular adhesion molecule-1 (VCAM-1), E-selectin and intracellular adhesion molecule-1 (ICAM-1), T-helper cells and endothelial macrophages are employed in the arterial endothelium. These macrophages phagocytose and oxidize the low-density lipoproteins to eventually result in the formation of foam cells via the induction of endothelial lesions. Post the foam cell creation, Thymocytes promote the formation and development of plagues via release of cytokines viz., IL-9, 11 and 12. Eventually, foam cells and plaque result in the formation of atheroma or atherosclerotic plaque (123, 124). The immune checkpoints, PD-1, PD-L1 and CTLA4 are inversely correlated with plaque formation and thus inhibition of the immune checkpoints results in enhanced recruitment of thymocytes which drive the development of plaque via induction of endothelial lesions (125, 126). This is also reflected in higher incidence (almost 4 to 5 fold) of cardiovascular adverse events viz., coronary revascularization, myocardial infarction and ischemic stroke, in cancer patients being administered immune checkpoint inhibitors (127). Experiments in animal models have shown that myeloid cells that are deficient in the expression of PD-1, promote the genes implicated in cholesterol synthesis and attenuate the genes involved metabolism of cholesterol, thus amplifying cholesterol levels, which is an established determinant of atherosclerosis (128). A diagrammatic representation of vascular injury leading to pro-atherogeneic activity due to inhibition of PD-1/L1 is shown in **Figure 2**.

## 6 Understanding the immune checkpoint-mediated atherosclerosis

Atherosclerosis is a major cardiovascular condition that contributes to the development of heart complications, leading to increased morbidity and mortality. It is primarily a chronic inflammatory disease of the arteries, marked by the accumulation of lipids in plaques (129, 130). The progression and destabilization of these plaques are significantly influenced by both innate and adaptive immunity. After endothelial injury, monocytes infiltrate the arterial walls and differentiate into macrophages. These macrophages uptake lipids and secrete pro-inflammatory cytokines, further aggravating the inflammation (131). Antigen-presenting cells, such as dendritic cells, are also recruited to the lesion site, where they activate Thymocytes, amplifying the inflammatory response. Effector Thymocytes, in turn, produce excessive pro-atherogenic cytokines, which increase plaque size and promote its destabilization (132).

**Figure 3:**
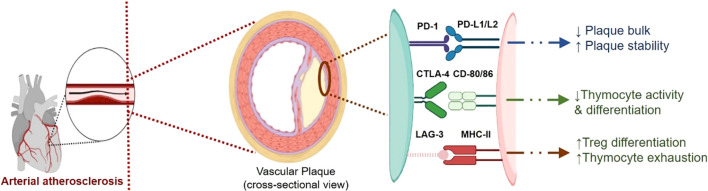
A digrammatic representation of involvement of different checkpoints viz., PD-1/L1, CTLA-4 and LAG-3, in atherosclerosis. The immune checkpoints imparts cardio-protective role and helps in maintaining cardio-homeostasis. These immune checkpoints imparts plaque stability and reduce the proliferation of plaque by controlling inflammation. Inhibition of these checkpoints disrupts this balance and thus promoting plaque development and proliferation.

Immune checkpoints, both co-stimulatory and inhibitory, regulate Thymocyte activation and play a crucial role in controlling the inflammatory response in atherosclerosis. The activation of these checkpoints can either stimulate or suppress Thymocyte function, influencing the development and growth of atherosclerotic plaques (133). Different immune checkpoints influence and drive the evolution and growth of plaque through their distinct pathway (**Table 1**). A digrammatic representation of involvement of different checkpoints in atherosclerosis is shown in **Figure 3**.

**Table 1:** Changes in immune checkpoints and other immune cells in atherosclerosis.

S. No.	Immune checkpoint inhibitor	Immunological changes in atherosclerosis	References
1.	PD-1/L1	↑ Macrophages, CD8+thymocyte and CD4+ thymocyte	[134]
		↑ Serum levels of TNF-α	
		↑ Cytotoxic effect of CD8+thymocyte	
		↑ Atherosclerotic leisions	
2	CTLA-4	↓ Effector CD4+ thymocyte	[135]
		↓ CD80, CD28 and CD86 expression on dendritic cells	
		↓ Atherosclerotic leisions and accumulation of CD4+thymocyte and macrophages in the plaque	
3	LAG-3	↑ T helper cells, memory thymocyte, Treg cells	[136]
		↑ T cell density in plaque	
		↔ No change in size of plaque	
4	TIM-3	↑ circulating monocytes and macrophages in leisions	[137]
		↑ CD4+ thymocyte and their activation	
		↓ Treg cells	
5	TIGIT	↑ Fatty streaks and atherosclerotic plaque	[138]
		↓ thymocyte in spleen and their activation	
		↑ Dendritic cells and their activation	
		↓ IL-10 in blood and spleen	
		↔ No effect on atherosclerotic plaque, collagen and macrophages	

Note: In this table, “↑” indicates upregulation or increase, and “↓” indicates downregulation or decrease.

Several immune checkpoint pathways are involved in the progression of atherosclerosis, offering potential therapeutic targets for managing this disease. For instance, inhibition of immune checkpoints like PD-1 and CTLA-4 has shown promise in reducing inflammation and stabilizing plaques, though caution is needed due to the potential for immune-related adverse effects. Targeting these pathways may offer a dual benefit: reducing plaque progression and minimizing cardiovascular events, while avoiding excessive immune activation that could lead to other complications. Further research into these therapeutic strategies, particularly the use of immune checkpoint inhibitors in conjunction with traditional cardiovascular treatments, is needed to fully understand their potential in managing atherosclerosis.

### 6.1 Involvement of PD-1/PD-L1 in atherosclerosis

Analysis of clinical investigation on immune checkpoint inhibitors has shown that there is a higher chance of developing cardiovascular complications viz., atherosclerosis, stroke and myocardial infarction (127, 139, 140). In fact, one of the studies has shown that almost 3-fold increase in the occurrence of atherosclerosis in human participants being administered immune checkpoint inhibitors (127). These findings lead to the assumption that thymocyte inhibition exerts a cardiovascular protective effect and thus reduces the chances of developing atherosclerosis. Thymocyte stimulation and growth is inhibited after interaction of PD-L1/PD-L2 with the corresponding PD-1 found on thymocytes. Activated Thymocytes exhibit inflammatory pro-atherogenic activity. Thus, PD-L1/PD-1 binding is inversely related to the pro-atherogenic activity.

This was confirmed in an animal study wherein knockout animals lacking PD-L1 or PD-L2 exhibited high atherosclerotic load along with elevated levels of CD4^+^ and CD8^+^ thymocytes. These animals also had amplified levels of Tumor Necrosis Factor (TNF)-α and amplified cytotoxicity of CD8^+^ thymocytes. Also, antigen-presenting cells from these knockout animals were found to have higher *in-vitro* efficiency in activating CD4^+^ thymocytes, in the presence as well as unavailability of cholesterol loading. Thus, the absence of PD-L1/PD-L2 was linked with a higher amount of plaque formation confirming the pivotal role played by PD-1/PD-L1 immune checkpoints in modulating plaque formation (141, 142).

Cytokine interferon (IFN)-γ, primarily produced by T-helper cells (Th cells) is a determinant in promoting atherosclerosis by deploying macrophages and thymocytes, promoting cytokine release and increase in endothelial antigen-presenting cells. Blocking or reduced expression of IFN-γ has been found to reduce atherosclerotic plaque size (143, 144). The linkage between PD-1 and PD-L1 results in differentiated Treg cells which interferes with the production of IFN-γ and TNF-α by the Th cells (145, 146). The stability of plaque is also influenced by IFN-γ. A stable atherosclerotic plaque reduces the risk of plaque breakdown and its subsequent dislocation, which may induce other cardiac complications like myocardial infarction and stroke. The cytokine IFN-γ can destabilize the plaque by hindering the propagation of vascular smooth muscles and the synthesis of cholesterol. Conversely, Treg cells express multiple immune checkpoints and promote the creation of anti-inflammatory cytokines viz., IL-10 and TGF-β, as they inhibit differentiation of inflammatory effector thymocytes. Thus, Treg cells exert and promote anti-atherogenic activity (147). This is confirmed in a study wherein attenuation in the population of Treg cells, deficit in IL-10 and interruption of TGF-β promote atherogenesis and can even worsen plaque formation (148, 149). Persistent exposure to inflammation and antigens can result in thymocytes exhaustion wherein these cells lose their function. This ensues reduction in the propagation of thymocytes, the release of cytokines and enhanced repressive expression of immune checkpoints like, LAG-3 and PD-1 (150). Exhausted thymocytes that express PD-1 are found in the atherosclerotic lesions. Consequently, inhibiting PD-1 by immune checkpoint inhibitors can reactivate the exhausted thymocytes, which can intensify and facilitate atherosclerosis (126).

Endothelial cells express PD-L1 under the influence of IFN-γ and TNF-α (151). This was confirmed in a study wherein PD-L1 was expressed by human umbilical cord vein endothelial cells (HUVECs) under the influence of IFN-γ (152). Constitutively HUVECs do not express PD-L1 (153), however, under the influence of IFN-α, β, γ and TNF-α HUVECs express PD-L1 (154). This indicates the critical role played by cytokines, IFN-α, β, γ and TNF-α, in promoting PD-L1 in the endothelial cells. Vascular endothelial growth factors (VEGF) also critically influence the appearance of PD-L1 in endothelial cells (155). It is well established that for cancer cells that exhibit PD-L1, protection from the cytotoxic and apoptotic effect of IFN-γ occurs by interfering with the JAK/STAT3/caspase7-dependent pathway (156, 157). Lymphatic endothelial cells expressing PD-L1 exhibit a similar pattern that helps them to prevent apoptosis (158). Thus, endothelial cells expressing PD-L1 exhibit the potential to counter the immune reaction by downregulating the CD8^+^ thymocytes and amplifying the activity of Treg cells, thus protecting endothelial cells from the pro-atherosclerotic effects (159).

### 6.2 Involvement of CTLA-4 in atherosclerosis

CTLA-4 immune checkpoints have been found to facilitate the thymocyte-mediated inflammatory development of atherosclerosis. Thymocyte stimulation is negatively controlled by CTLA-4. This was confirmed in animal studies that were devoid of CTLA-4 wherein such animals exhibit enlarged lesions in the aorta. Also, the animal receiving antibodies blocking CTLA-4 exhibits an almost two-fold increase in plaque size and area with elevated Thymocyte and macrophage amounts in these plaques along with reduced collagen and smooth muscle cell content (160). Contrary to this study, animals that overexpress CTLA-4 or are administered an analogue of CTLA-4 decreased the population of CD4+thymocyte, reduction in cellular differentiation and generation of pro-inflammatory cytokines (IL-10 and IF-γ) and reduction (~58.5%) in thickness of the intima (135, 161). At the cellular level, induced pluripotent stem cell-derived cardiomyocytes (iPSC-CM), that were exposed to hypoxia which simulates the ischemic cardiac injury post-myocardial infarction, exhibit amplified amounts of CD80 and CD86 at the genetic level. The appearance of CD80 and CD86 at protein and genetic levels is increased post-cardiac injury (162). Amplification in the expression of CTLA-4 results in a drop in lesional CD4+ thymocytes. Also, a reduction in effector thymocytes in lymphatics due to disability of thymocytes to differentiate and grow under the conditions of reduced cytokine levels (135).

Intracellular dendritic cell binding of CTLA-4 and CD80/86 incites expression of Indeolamine 2,3-dioxygenase (IDO) (163). Augmentation in IDO results in halting thymocyte proliferation resulting in reduced activation of thymocytes and enhanced apoptosis of thymocytes. As a result, treatment with anti-CTLA-4 results in alleviating CD80/86 interaction which reduces the self-tolerance (164, 165).

### 6.3 Involvement of LAG-3 in atherosclerosis

The LAG-3, expressed on thymocytes, is the ligand that binds with MHC II (166). Clinical studies have shown that immune checkpoint LAG-3 has not been found to be correlated with cardiac complications like atherosclerosis, and coronary heart disease, because of which the FDA has approved them for anti-tumor therapy. Although long-term follow-up evaluation data is lacking. However, an independent relationship between LAG-3 and coronary heart disease has been reported and atherosclerotic plaque expresses LAG-3 in exhausted Thymocytes. LAG-3 is a potential predictive tool as a determinant of coronary heart disease and an indicator of plasma high-density lipoprotein-cholesterol (150, 167). The function of LAG-3 was evaluated in bone marrow chimeras hematopoietic cells devoid of LAG-3 and knockout mice devoid of LAG-3. The LAG-3 deficiency, as well as treatment with anti-LAG-3 antibodies, resulted in an enhanced population of memory cells and Thymocytes reducing IFN-γ, which was balanced with an amplification in Treg cells. Consequently, the plaque size remains unaffected, although there was an amplification in the stimulated thymocyte population in the plaque (136). LAG-3 and PD-L1 both aid in enhancing the stability of endothelial plaques (159).

Apart from the above immune checkpoint functionality in atherosclerosis, self-antigens also contribute to the development of plaque. A self-antigen, keratin-8 was found to increase the levels of PD-1 appearance in the human peripheral blood mononuclear cells (PBMCs) as against PBMCs obtained from coronary artery disease patients. This finding suggests that keratin-8 can potentially stimulate PD-1 expression thus restricting the thymocyte reaction and imparting protective function in anomalous patients of coronary artery disease (168, 169). Further, reports suggest that gastric adenocarcinoma cells with PD-L1, exhibit higher intracellular intake of lipids via fatty acid binding protein (Fabp4/5) elevation. Similar activity is exhibited by PD-L1-expressing cells. Consequently, interferance with PD-L1 results in reduced Fabp4/5 protein level and decreased intracellular intake of lipids. This results in higher availability of free lipids at the plaque site causing aggravation of atherosclerosis (170).

## 7 Understanding the immune checkpoint-mediated myocarditis

Myocarditis, an inflammation of the myocardium caused by infectious or non-infectious agents, can lead to severe complications, including heart failure and dilated cardiomyopathy (171). Although relatively rare, myocarditis is an important cause of heart failure and can manifest clinically through a range of symptoms such as palpitations, cardiogenic shock, arrhythmias, and heart failure (172–175). Chronic inflammation in myocarditis can lead to myocyte necrosis, fibrosis, and scarring, ultimately contributing to the development of dilated cardiomyopathy (174).

Recent clinical studies have emphasized the growing concern of myocarditis associated with immune checkpoint inhibitors (ICIs). A retrospective analysis found that 50% of deaths related to immune checkpoint inhibitors were due to myocarditis (17). This condition can be fulminant, leading to cardiogenic shock, ventricular arrhythmias, and, in severe cases, cardiac arrest (176). The frequency of myocarditis is notably higher with PD-1/PD-L1 pathway inhibition compared to CTLA-4 blockade, with incidence rates of 0.41% and 0.07%, respectively (17). Recent studies have also shown that patients receiving PD-1/PD-L1 inhibitors have a higher risk of developing myocarditis (69.1% of cases), compared to those treated with CTLA-4 inhibitors (20%) (177, 178).

Furthermore, combination therapies involving PD-1/PD-L1 inhibitors and other immune checkpoint modulators, such as LAG-3 inhibitors, have led to an even higher incidence of myocarditis, with up to 1.7% of patients experiencing this adverse event, compared to 0.6% with single-agent PD-1/PD-L1 therapy (179). Moreover, there have been several reports of myocarditis occurring in patients receiving combination therapies of ICIs with radiotherapy or chemotherapy, exacerbating the risk of cardiotoxicity (180–183).

**Figure 4:**
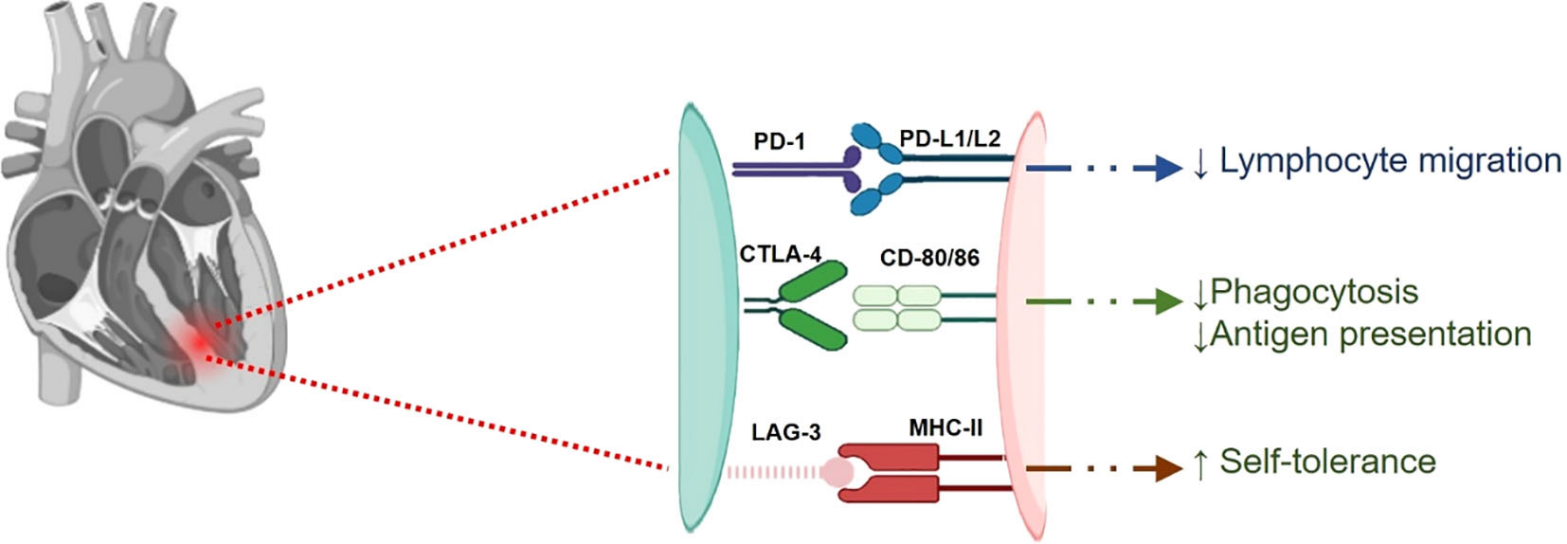
A digrammatic representation of involvement of different checkpoints viz., PD-1/L1, CTLA-4 and LAG-3, in myocarditis. PD-1/L1 and CTLA-4 are responsible for keeping autoimmunity in check and thus reducing cardiac inflammation. These immune checkpoints reduce lymphocyte infiltration and phagocytosis. Inhibition of these checkpoints disrupts this balance and thus resulting inflammatory conditions leading to myocarditis.

**Figure 5:**
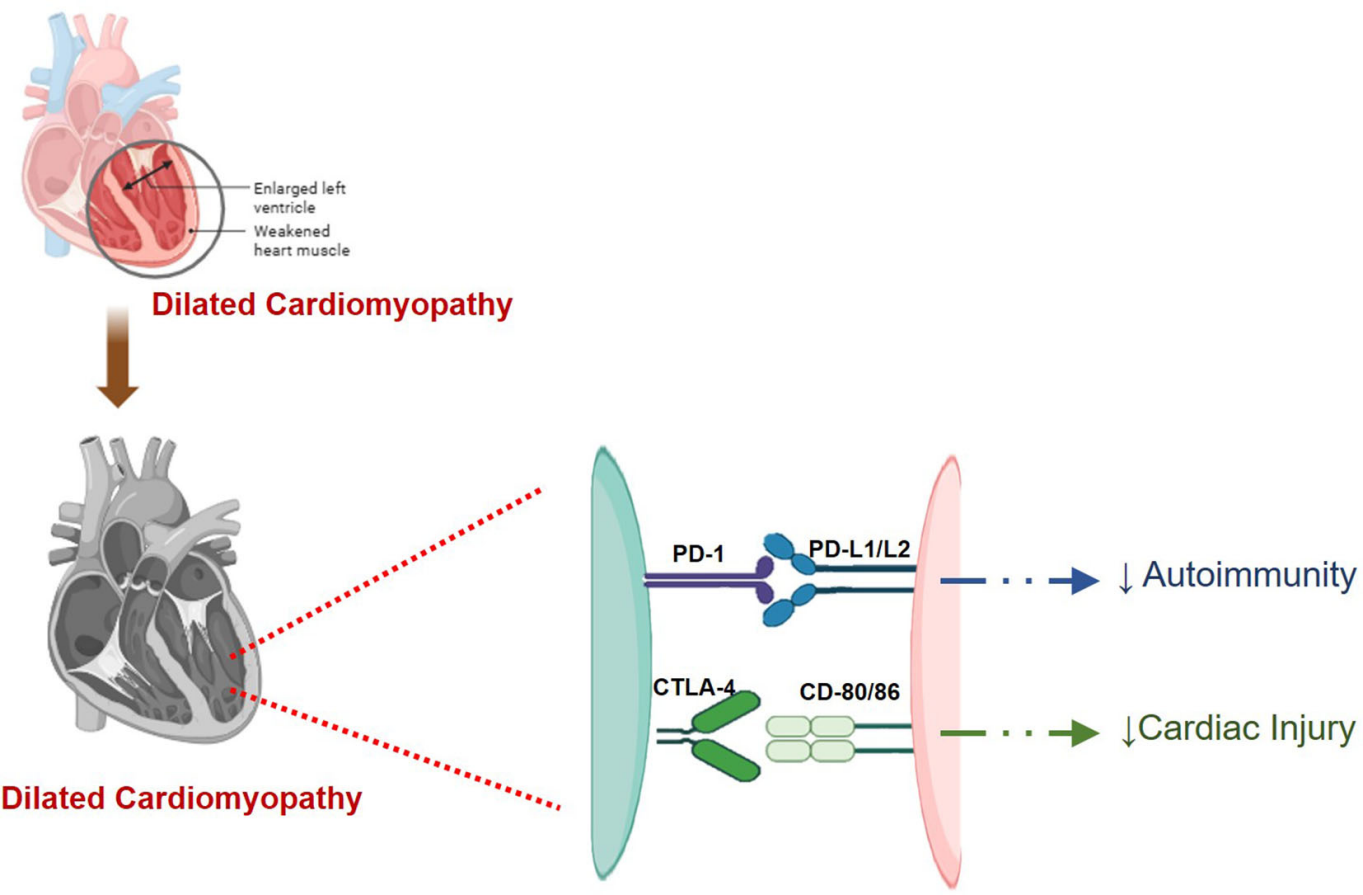
A digrammatic representation of involvement of different checkpoints viz., PD-1/L1, and CTLA-4 in cardiomyopathy. PD-1/L1 and CTLA-4 reduce cardiac injury and autoimmunity in check. Inhibition of these checkpoints disrupts this balance and thus resulting in cardiomyopathy.

These findings highlight the critical need for careful monitoring and management of cardiac health in patients receiving immune checkpoint inhibitor therapies, particularly in combination with other treatments. A diagrammatic representation of the involvement of different immune checkpoints, including PD-1/PD-L1, CTLA-4, and LAG-3, in myocarditis is shown in **Figures 4**, **5**.

### 7.1 Involvement of PD-1/PD-L1 in myocarditis

A lack of harmony between self- tolerance and autoimmunity was the basic reason for the induction of immune checkpoint inhibition induced myocarditis (184). Although the exact relationship between myocarditis and immune checkpoints is not exactly established, however, certain studies suggest the influence of immune checkpoints on myocarditis. Clinical evaluation of patients of myocarditis, post-treatment with CTLA-4 and PD-1/PD-L1 inhibitors therapy, showed infiltration of thymocytes in the myocardial and skeletal cells (185). Enhanced activation and relocation of thymocytes in cardiac cells along with higher presentation of antigen and phagocytosis is observed when treated with inhibitors of PD-1/PD-L1 and CTLA-4 (186).

In a study, in mice with cardiomyopathy, cardiomyocytes expressed PD-L1 which interfered with and halted the thymocytes act by negatively controlling the production of IFN-γ (187). Immune checkpoint expression on the endothelial cells and cardiomyocytes seems to exert a protective function against induction of myocarditis and injury. This was confirmed in a study wherein PD-L1 and PD-L2 were found to be amplified under the influence of IFN-γ, in animals with CD8+thymocyte -moderated myocarditis. Interference with the production of IFN-γ as well as haltering or devoid of PD-L1/L2, thus exacerbated the disease condition (62, 188). Animal studies also show that the expression of PD-L1/PD-L2 was observed in endothelial cells that have been injured by the inflammation in myocarditis. Interestingly, endothelial cells of control animals did not exhibit PD-L1/L2 (154). These findings collectively indicate the protective function of PD-L1/PD-L2 in myocarditis (189, 190). Studies suggest that cardiac endothelial cell and cardiomyocyte apoptosis is inhibited by the intracellular PD-L1 as well as MHC-II signaling through MAPK/Erk and PI3K/Akt pathways (156, 157, 166).

### 7.2 Involvement of CTLA-4 in myocarditis

A genetically modified animal model, wherein the animals were devoid of PD-L1/L2 completely while haploinsufficiency for CTAL-4 was present. The animals completely devoid of PD-1/L1 and haploid status for CTLA-4 were found to exhibit a high rate (~50%) of mortality due to myocarditis. Treatment of such animals with CTLA-4 inhibitor improved the survival of the animals along with the reduced infiltration of thymocytes at the cardiac injury site. Instead, animals devoid of PD-1/L1 but with homoallelic CTLA-4 did not develop myocarditis. This clearly indicates that PD-1/L1 and CTLA-4 interact together at the genetic level for the manifestation of myocarditis (191).

Giant cell myocarditis has been related to CTLA-4 inhibitors. Giant cell myocarditis is a deadly condition wherein acute dilated cardiomyopathy along with ventricular tachycardia and eventually cardiac block occurs (192). Histologically, in giant cell myocarditis Thymocyte mediated necrosis of myocytes and macrophages acquired multi-nucleated giant cells are formed (193). Giant cell myocarditis is chiefly a condition driven by CD4+Thymocytes and chemokines viz., C-X-C Motif Chemokine Receptor 3 (CXCR3). The CXCR3 is vital in the pathways, involved in the diversification, activation and deployment of CD4+ thymocytes, like, MAP kinase and PI3K/Akt pathways. CXACR3 is involved in the activation and deployment of CD4+ thymocytes and has not been found to be involved in the CD8+thymocytes. The upregulation of CD8+ thymocytes is primarily interrelated with immune checkpoint inhibitors and not CXACR3 expression. Inhibition of CTLA-4, however, has been found to upregulate the appearance of CXACR3, which in turn incites giant cell myocarditis (194).

### 7.3 Involvement of LAG-3 in myocarditis

Although LAG-3-associated myocarditis is rarely seen, few cases of myocarditis have been reported when administered with anti-LAG-3 agents. In the RELATIVITY-047 clinical trial carried out on more than 700 subjects, 1.7% of the subjects reported developing myocarditis when administered a blend of relatlimab (LAG-3–blocker) –nivolumab (PD-1 inhibitor) as against 0.6% of the subjects that received monotherapy of nivolumab only (179). Studies in knockout animal models that are devoid of LAG-3 also point out to lack of relation between LAG-3 and myocarditis (195). However, studies in the knockout model that was devoid of LAG-3 as well as PD-1, exhibited the development of myocarditis along with elevated levels of TNF-α and excessive infiltration of Thymocytes, though Treg cells exhibited a controlled functioning in such animals (196).

The alternative proposed mechanism to induce myocarditis encompasses the presence of shared cardiac and tumor antigens for cells expressing PD-L1 viz., endothelial cells and myocytes. Another proposed mechanism includes the existence of a pre-existent immune response that induces autoimmunity, like higher levels of anti-troponin T antibodies in patients developing myocarditis post PD-1 inhibitor therapy (197). Similarly, a PD-1 and CTLA-4 inhibitor treatment-induced rhabdomyolysis polymyositis patient exhibited higher levels of antibodies against striated muscles (198).

## 8 Understanding the immune checkpoint-mediated cardiomyopathy

Animal models deficient in PD-1 have provided significant insights into the development of cardiomyopathy. These animals exhibited compromised cardiac contractile function, leading to untimely mortality. Notably, PD-1-deficient animals showed an accumulation of IgG on the surface of cardiomyocytes, along with circulating IgG antibodies targeting cardiac troponin I. However, no immune cell infiltration was observed, indicating that the mechanism behind cardiomyopathy in this model was primarily inflammatory rather than immune cell-driven (99, 119).

In human studies, PD-1 expression was found to be upregulated in the intercalated discs and myocardium of patients who had developed myocardial infarction and dilated cardiomyopathy. The expression of PD-1 in these patients was inversely correlated with left ventricular ejection fraction (LVEF) and directly associated with left ventricular end-diastolic volume, suggesting a role for PD-1 in regulating cardiac function during injury (199).

Furthermore, the upregulation of myocardial PD-L1 has been shown to mitigate cardiac injury by negatively regulating immune responses and reducing Thymocyte activity (200). A study on immune checkpoint inhibitor-induced dilated cardiomyopathy found that cardiomyocytes expressed PD-L1, which helped suppress the production of pro-inflammatory cytokines, including TNF-α and IFN-γ, ultimately reducing inflammation and tissue damage (187).

## 9 Other immune checkpoints viz., T-cell immunoglobulin and mucin domain 3 (TIM-3) and T-cell immunoglobulin and ITIM domains (TIGIT) etc.

Associated cardiovascular complications, other immune checkpoints are alos being evaluated. These immune checkpoints include TIM-3 or Hepatitis A virus cellular receptor 2 (HAVCR2), V-set and transmembrane domain-containing protein 3 (Vstm3), TIGIT or Washington University cell adhesion molecule (WUCAM) and V-Set and immunoglobulin domain-containing protein 9 (VSIG9) (201). These new immune checkpoints can be utilized as novel targets for the treatment of tumors and are currently being clinically evaluated, alone or in conjunction with PD-1/L1 inhibitors or CTLA-4 inhibitors (202–217).

Immune checkpoint inhibitor, TIM-3 or HAVCR2, is part of TIM family and can bind to four corresponding ligands viz., Galectin-9 (LGALS9), high mobile protein B1 (HMGB1), phosphatidylserine (PtdSer) and surface-bound ligands carcinoembryonic antigen cell adhesion molecule 1 (CEACAM-1) (67, 218–221). Under the influence of IFN-γ, CD4+thymocytes and CD+8 thymocytes amplify the expression of TIM-3 (204, 205). TIM-3, expresses on NK cells, Treg cells, Th17, dendritic cells and macrophages and inhibits cytokines IFN-γ and TNF, thereby suppressing type I immune response (222–232). Although some reports suggest that the TIM-3 pathway serves both stimulatory as well as inhibitory function (223). Structurally, TIM-3 possesses five tyrosine conserved residues, of which phosphorylation of Tyr 265 and Tyr272 is essential in humans. Interaction of TIM-3 with HLA-B-associated transcript 3 (Bat3) and Lymphocyte-Specific Protein Tyrosine Kinase (LCK) on the lipidic rafts to promote differentiation and survival of thymocytes (233–235). However, binding with CD148 and CD45 results in replacement of BAT-3 on TIM-3 and the deploying tyrosine phosphatase causing inactivation of LCK. This inhibits the proliferation of thymocytes by negatively regulating the signaling of Thymocyte receptors (235, 236). Thus, the inhibitory action of TIM-3 on Thymocytes is mediated by BAT-3. Clinical evaluation of the inhibitors of TIM-3 are underway and only few cases of cardiovascular complications has been reported. Infact, some report suggest that atherosclerosis is inversely related to TIM-3. The study findings show that TIM-3 inhibitors amplify the atherosclerotic lesions which is accompanied by enhanced levels of macrophages, CD+4 Tcells and monocytes, along with diminished levels of Treg cells and B cells (137). Another study evaluated the effect of co-treatment with PD-1 and TIM-3 inhibitor and it was found that cytokines which exert anti-atherogeneic effect were reduced but TNF-α and IFN-γ levels were amplified resulting in advancement of atherosclerosis (237).

Another immune checkpoint, TIGIT, or Vstm3/VSIG9/WUCAM, is categorically expressed on lymphocytes, specifically on NK cells, effector CD8+ thymocytes, regulatory CD4+ thymocytes cells and helper thymocytes (207, 238–241). TIGIT expression is amplified especially on Treg cells in the tumor microenvironment (242–244). TIGIT regulate the NK cells and thymocytes functioning via binding with CD155 or poliovirus receptor or Necl-5. TIGIT exhibit a lower affinity toward another nectin family receptor, CD112 or poliovirus receptor related-2 or Nectin-2 (245–248). TIGIT interferes with the proliferation and activation of CD8+thymocytes by exerting its effect on thymocyte receptor expression. This incites negative regulation of the thymocyte receptor α chain and other parts of the receptors (249, 250). TIGIT can suppress the cytotoxic activity and differentiation of CD8+ thymocyte cells via p-ERK signaling (251, 252). By competitively interacting with CD226 or DNAM-1, thymocyte stimulation can be reduced (253). Clinical evaluation of TIGIT inhibitors has so far not reported any cardiovascular complications and additional studies are desirable to establish the safety of TIGIT inhibitors (254–261).

## 10 Clinical implications of inhibition of immune checkpoints

Cardiac implications are often overlooked in the clinical evaluation of immune checkpoint inhibitors (262–265). Inhibition of immune checkpoints has been found to induce myocarditis, vasculitis, pericardial disease and even heart failure (266). A brief overview of clinical implications of different immune checkpoints are enlisted in **Table 2**. Nevertheless, different studies have shown that animals devoid of PD-1 or administered with an inhibitor of CTLA-4 exhibit inflammation in the cardiac tissues (93, 99, 186, 275–278). Amplification of PD-L1 in animals with myocarditis has been reported (120, 279, 280). BALB/c mice devoid of PD-1 developed autoimmune cardiomyopathy (99). Inhibition or negatively regulating PD-1/PD-L1 has been found to amplify atherosclerosis in animals (142). Also, in such animals, troponin was found to be the cardiomyopathy-inducing antigen in animals devoid of PD-1 (119).

**Table 2:** Cardiovascular complications associated with different PD-1 and CTLA-4 inhibitors and status post- treatment.

S. No	Immune check point inhibitor & (Use)	Immune checkpoint target	Cardiovascular complication	Status of cardiovascular condition post treatment	Reference
1	Ipilimumab, *(Melanoma)*	CTLA-4	Myocardial fibrosis	Reduced	[267]
			Cardiomyopathy	Diuretics resulted in positive effect	[94]
			Left ventricular dysfunction	β blocker and ACE inhibitors resulted in positive effect	[268]
			Myocarditis	Reduced	[94]
			Heart failure	Long term lowering in ejection fraction, with diuretics as well	[94]
			Myocarditis along with congestive heart failure	Steroids, β blocker and ACE inhibitors resulted in positive effect	[94]
2	*Pembrolizumab, (Melanoma, urothelial carcinoma, gastric cancer, Non- small-cell lung cancer (NSCLC), large B cell lymphoma, cervical cancer, Hodgkin’s lymphoma)*	PD-1	Myocarditis	Reduced	[269]
			Stable angina pectoris	Termination of Pembrolizumab helps to reduce	[269]
			Sinus tachycardia	Treatment with metoprolol succinate helps to reduce	[269]
			Heart failure	Treatment with steroid, β blocker, AT_2_ receptor blocker, diuretics and aldosterone helps to reduce	[270]
			Cardiac arrest	Treatment with steroids, defibrillation, and catecholamines helps to resolve it	[94]
3	*Nivolumab (Melanoma, NSCLC, Hodgkin’s lymphoma, SLCL, head & neck cancer RCC, metastatic colorectal cancer, HCC, urothelial carcinoma)*	PD-1	Myocarditis (in melanoma)	Treatment with prednisolone helps to reduce	[271]
			Asystolia	Termination of Nivolumab along with resuscitation and treatment with prednisolonehelps to reduce	[269]
			Myocarditis (in NSCLC)	Treatment with amiodarone and glucocorticoids or steroid, β-blockers, diuretics and ACE inhibitors, helps to reduce	[94]
4	Ipilimumab + *Nivolumab (colorectal cancer, melanoma, RCC)*	PD-1 + CTLA-4	Myocarditis	Treament with steroids helps to reduce	[180, 272]
			Myocarditis and cardiomyopathy	Treament with steroids helps to reduce	[273]
			Ventricular arrhythmia	Can be reduced with treatment	[94]
			Smouldering myocarditis	Treatment with corticosteroid therapy helps to reduce	[274]

Such cardiac complications have also been observed in human subjects undergoing clinical evaluation for immune checkpoint inhibitors (281). One such case of myocardial fibrosis was observed in a multi-center clinical evaluation of ipilimumab (267). Similarly, another case of autoimmune myocarditis that prompted heart failure was observed in subjects undergoing treatment for melanoma with pembrolizumab (270, 282). Ipilimumab instigated the late development of pericarditis in a patient undergoing treatment for melanoma (283). Several cases of cardiotoxicity were observed when administered ipilimumab and/or nivolumab/pembrolizumab in a multicenter clinical study. In fact, a couple of mortalities were also reported even though subjects were given treatment for the cardiac complications. Also, five cases develop myocarditis among eight cases of cardiac complications when administered inhibitors of PD-1 and/or CTLA-4 (94). In a small clinical evaluation, the administration of ipilimumab, in subjects having a history of cardiovascular disease or autoimmune condition, resulted in the exacerbation of autoimmune complications in almost 50% of subjects (284). Fatal fulminant myocarditis was reported in two subjects when administering a blend of ipilimumab and nivolumab in subjects suffering from melanoma. Both the subjects had a pre-existing condition of hypertension. Apart from these two cases, 18 cases out of 20594 subjects were reported to have developed myocarditis (94). In fact patients receiving a combination exhibit a severe and higher frequency of myocarditis (0.27%) as against those receiving nivolumab alone (0.06%) (185). In participants having coronary heart disease, levels of PD-L1 expressed in Treg cells were inversely related to the severity of coronary heart disease and has the potential to be utilized as a biomarker for it (285).

B-type natriuretic peptide (BNP) and troponin have been found to increase in case of immune checkpoint inhibitor-induced cardiotoxicity and can be utilized as biomarkers of cardiotoxicity (185, 270, 286, 287). Another potential biomarker for cardiotoxicity is anticonductive tissue autoantibodies (ACTA) (288, 289).

## 11 Immune checkpoint inhibitors mediated cardiovascular complications leading to heart failure

A retrospective analysis revealed that of the 424 subjects who received immune checkpoint inhibitors, 14.6% of them (62 subjects) reported cardiovascular condition. Further, of these 62 subjects, 5.6% had heart failure with monotherapy of immune checkpoint inhibitor while 6.1%, receiving combination or dual therapy of immune checkpoint inhibitor reported to have heart failure (290). Similar outcome was reported in meta-analysis of 13346 subjects, 3.1% of the subjects receiving either CTLA-4 inhibitor or PD-1/L1 inhibitor and 5.8% of the subjects receiving combination therapy reported to have heart failure. Interestingly, addition of chemotherapy along with immune checkpoint inhibitor did not significantly added to the incidence of heart failure (3.7%) (178). Further, administration of CTLA-4 inhibitors seems to induce higher frequency of cardiovascular complications viz., myocarditis, pericarditis etc. (94, 286, 291–296). Administration of PD-1/L1 inhibitors like, nivolumab and pembrolizumab etc. have been reported to cause cardiotoxicity like myocarditis (197, 270, 274, 297–310). These findings were further consolidated by the observation of a meta-analysis of data of 32518 subjects that immune checkpoint inhibitors can induce or amplify the risk of cardiotoxicity viz., myocardial infarction, myocarditis, pericarditis, cerebral arterial ischemia, dyslipidemia and heart failure. In fact heart failure is the most severe cardiotoxicity observed with treatment of immune checkpoint inhibitors (311). Thus, immune checkpoints are key determinants of cardiac homeostasis and alteration or blocking of the immune checkpoint can severely affect cardiac health (312–318).

## 12 Conclusion

Immune checkpoints are essential components of the immune system that help maintain self-tolerance and prevent autoimmune damage, particularly in critical organs such as the heart, eyes, brain, and kidneys. In the heart, immune checkpoints regulate cardiac homeostasis by controlling immune responses that can otherwise lead to cardiovascular complications, including atherosclerosis, myocarditis, and coronary heart disease, which can progress to heart failure. Clinical reports have highlighted the risks of cardiac injury and cardiotoxicity associated with immune checkpoint inhibition, particularly with the use of PD-1/L1 and CTLA-4 inhibitors. The development and progression of atherosclerosis have been found to be exacerbated by these inhibitors, while the role of LAG-3 in cardiotoxicity is still under investigation. Inhibition of the PD-1/PD-L1 pathway, in particular, has been shown to pose a higher risk for inducing myocarditis compared to CTLA-4 inhibition. The combination of PD-1/PD-L1 inhibitors with TIM-3 inhibition has also been linked to an increased incidence of atherosclerosis. These findings underscore the critical need for further understanding of immune checkpoint regulation in cardiac homeostasis.

On the other hand, modulating immune checkpoints presents a promising strategy for targeting specific stages or cell types involved in cardiovascular diseases, such as atherosclerosis and myocarditis. Given that immune checkpoints operate through both co-stimulatory and inhibitory pathways, selectively manipulating these pathways could lead to therapeutic benefits. Additionally, alterations in the levels of immune checkpoints and their ligands may serve as predictive biomarkers for cardiotoxicity. Future research should focus on identifying safe and effective strategies for regulating immune checkpoints in order to develop novel therapeutic and prognostic tools for managing cardiovascular health. Further clinical trials and preclinical studies are necessary to explore the long-term effects of immune checkpoint modulation on cardiovascular diseases and to refine therapeutic approaches that minimize the risk of cardiotoxicity.
